# Iso-mukaadial acetate and ursolic acid acetate bind to *Plasmodium Falciparum* heat shock protein 70: towards targeting parasite protein folding pathway

**DOI:** 10.1186/s13065-024-01159-6

**Published:** 2024-03-18

**Authors:** Francis Opoku, Penny Govender, Addmore Shonhai, Mthokozisi BC Simelane

**Affiliations:** 1 Department of Chemical Sciences (formerly Department of Applied Chemistry), University of Johannesburg, Doornfontein Campus, P.O. Box 17011, Johannesburg, 2028 South Africa; 2 Department of Chemistry, Kwame Nkrumah University of Science and Technology, Kumasi, Ghana; 3 Department of Biochemistry & Microbiology, University of Venda, Thohoyandou, South Africa; 4 Department of Biochemistry, University of Johannesburg, Auckland Park, South Africa

**Keywords:** Heat shock protein, Inhibitor, iso-mukaadial acetate, Ursolic acid acetate, *Plasmodium Falciparum*

## Abstract

*Plasmodium falciparum* is the most lethal malaria parasite. *P. falciparum* Hsp70 (*Pf*Hsp70) is an essential molecular chaperone (facilitates protein folding) and is deemed a prospective antimalarial drug target. The present study investigates the binding capabilities of select plant derivatives, iso-mukaadial acetate (IMA) and ursolic acid acetate (UAA), against *P. falciparum* using an *in silico* docking approach. The interaction between the ligands and *Pf*Hsp70 was evaluated using plasmon resonance (SPR) analysis. Molecular docking, binding free energy analysis and molecular dynamics simulations were conducted towards understanding the mechanisms by which the compounds bind to *Pf*Hsp70. The molecular docking results revealed ligand flexibilities, conformations and positions of key amino acid residues and protein-ligand interactions as crucial factors accounting for selective inhibition of Hsp70. The simulation results also suggest protein-ligand van der Waals forces as the driving force guiding the interaction of these compounds with *Pf*Hsp70. Of the two compounds, UAA and IMA bound to *Pf*Hsp70 within the micromolar range based on surface plasmon resonance (SPR) based binding assay. Our findings pave way for future rational design of new selective compounds targeting *Pf*Hsp70.

**Supplementary Information:**

The online version contains supplementary material available at 10.1186/s13065-024-01159-6.

## Introduction

The continued emergence of malaria drug-resistant parasites continues to emphasize the need for the identification of new alternative molecules. Annually the *Plasmodium sp.* is responsible for approximately 400,000 deaths worldwide, with the *Plasmodium falciparum* being the most lethal causative agent of malaria. This apicomplexan parasite undergoes a complex life cycle due to its ability to transverse in multiple hosts and the constant need to adapt its mechanism to various physiological conditions. As such, it has been hypothesized that as part of its survival mechanism and strategy, the *P. falciparum* expresses a repertoire of heat shock proteins (Hsps) to facilitate its survival under sever physiological conditions such as the increased temperature changes it encounters as it shuttles from the cold-blooded mosquito vector to the warm-blooded human host, the increase in temperatures it encounters as a result of the malarial febrile episodes as well as oxidative stress [1, 2].

*P. falciparum* Hsps, such as *Pf*Hsp70 are ATP-dependent chaperones that are constitutively expressed to maintain cellular homeostasis under normal and stressful physiological conditions. These proteins play major roles such as the refolding of nascent or unfolded polypeptides, translation and translocation of proteins, and maintenance of cellular processes including protein assembly [3]. *Pf*Hsp70 is an essential cytosol localized protein that cooperates with other partner proteins or co-chaperones to facilitate protein folding processes and induce parasite development and pathogenesis. This protein is also said to be expressed throughout the parasite erythrocytic stage highlighting its significance in the development of the parasite.

Additionally, it has also been suggested as a possible cause of antimalarial drug resistance making it an ideal and prospective antimalarial drug target [4, 5]. This, therefore, justifies the importance of the pursuit of small molecular weight compounds against the protein. Zininga and co-workers (2017), reported the presence of two small inhibitors namely polymyxin B (PMB) and epigallocatechin-3-gallate (EGCC), and postulated that these compounds inhibit the chaperone activity of *Pf*Hsp70 by interfering with the proteins chaperone’s ability to interact with its known functional partners [6].

This is thought to be due to the binding of the compounds to the N-terminal ATPase domain of the protein which likely leads to the competition of the binding site between the protein substrates, adenosine triphosphate (ATP)/adenosine diphosphate (ADP) and the compounds. Furthermore, our previous studies have demonstrated that *Pf*Hsp70 is an efficient receptor for potential targeting using novel antimalarial compounds [7, 8]. However, there is a lack in computational techniques and determination of the binding strength of chemo-ligands towards the identified protein, yet this is crucial in the design of possible drug compounds [9].

Over the past decades, molecular docking has emerged as a powerful tool used to provide insights into protein-ligand interactions by studying, predicting, and modeling the interactions of small molecules within the active site of a target receptor or protein at an atomic level [10]. In addition, molecular docking can be used to infer the ADMET properties of drug candidates allowing for the prediction of the drug’s pharmacokinetic behaviour and toxicity profile [11]. Specifically, in the current study, we conducted molecular docking, Prime/Molecular Mechanics-Generalized Born Surface Area (MM-GBSA), and molecular dynamics (MD) simulations to analyze the interaction mechanisms, dynamic behaviors, binding affinity, and modes of the protein-ligand complex in a physical environment with solvent.

In our hands, Salomane et al. (2021) reported IMA and UAA as potential inhibitors of *Pf*Hsp70-1 chaperone activity [12], however, the nature of this interaction is yet to be fully elucidated. Thus, the current study aims to conduct an in-depth analysis of the interaction between the identified compounds and *Pf*Hsp70 using computational and lab-based assays.

## Materials and methods

### Molecular docking

The Hsp70 crystal structure was retrieved from the RCSB Protein Data Bank (PDB ID: 4J8F) [13]. 4J8F protein was selected as the inhibitor based on the search and screening results. It is a target protein that plays a central role in the cellular defence against toxic protein aggregation and for the maintenance of protein homeostasis and is a marker of malaria. Hsp70 has been shown to reduce pathologic protein aggregation in cellular models of Parkinson’s and Huntington’s disease. Hsp70 has proven useful in facilitating the proteolytic clearance of toxic, aggregation-prone proteins. Similarly, boosting Hsp70 might be advantageous in cancer therapy by accelerating the clearance of metastable, oncogenic mutant proteins. Moreover, 4J8F complex was prepared as the initial receptors for the docking studies by the Protein Preparation module implemented in Schrödinger Release 2019-2 [14]. This includes the addition of hydrogen atoms, removing non-bonded heteroatoms and all crystal water molecules, assigning partial charges and appropriate protonation states at pH = 7.0 and optimising the structure using the OPLS3e force field until the RMSD attained a value of 0.3 Å. The missing loop structures and side chains of 4J8F complex were added by using Prime module [15] in the Schrödinger Release 2019-2 [16]. The three-dimensional structures of iso-mukaadial acetate (IMA) and ursolic acid acetate (UAA) ligands were drawn in Maestro 11.8 suite [16]. Both ligands were processed with the ligprep tool [17] in Schrödinger Release 2019-2 to produce the most possible ionisation state of tautomer and enantiomer using Epik at pH = 7.0. The quantum mechanics/molecular mechanics (QM/MM) docking was performed using the OPLD procedure in Schrödinger to account for the receptor’s polarization of the ligand charge. In addition, hydrogen atoms and OPLS 2005 force field partial charges were added and assigned to ensure the rationality of the charges. Charges were estimated using QM calculations, and partial charges were substituted for each ligand complex. The 6-31G** basis sets and OPLS 2005 force field was utilized to perform single-point energy calculations and geometry optimization of structures. The QM region of all coordinates was free to adjust during the optimization.

The prepared proteins and ligands were docked using the Glide module in the Schrödinger Release 2019-2 suite [15] with the standard precision (SP) method to evaluate the binding pose of each compound in the Hsp70 binding sites. The scoring grid for docking was generated by enclosing the compound in a grid box of 28 × 28 × 28 and 34 × 34 × 34 points of dimension with the *x*, *y*, *z* coordinates of 26.220, 18.270, -19.00 and 32.250, 16.079, -28.584 for IMA and UAA, respectively, using the Receptor Grid Generation module. A grid box defined to cover the entire system with the same grid box size and dimensions is required to ascertain the probable ligand-binding location on the protein [18, 19]. However, the shape and properties of the receptors were represented on a grid by different grid box sizes and dimensions that progressively provided more accurate scoring to the ligand poses. Multiple scoring functions, such as docking score, glide score function, glide emodel, energy, Ecoul and Evdw term were used to choose the final best-docked structure. The glide score is the extended and modified version of the empirically based function [20]. Glide energy is the modified glide emodel and van der Waals (vdWs)-Coulomb interactions energy that combines strain energy, glide vdWs, coulombic and score of the ligand. The terms Ecoul and Evdw are the electrostatic interaction energy and vdWs interaction energy. Before using this technique on ligand-receptor complexes, we ran docking tests and generated several ligand conformations. During the docking step, we used Glide to create 100 initial poses and grouped them using a 1.5 Å RMSD threshold. Finally, we prepared ten different ligand poses for each ligand. After clustering, we scored and ranked the representative poses using Emodel. Here, energies of receptors, ligands, and complexes are estimated individually using the OPLS 2005 force field in a solvent environment, and the energy difference is then calculated. To score the poses, the top-ranking poses based on energy differential value were selected.

### Molecular dynamics (MD) simulations

MD simulations were conducted for both protein complexes using the Desmond module [15, 16] of Schrodinger Release 2019-2. Each system was firstly neutralised with chlorides and sodium ions and then immersed in an orthorhombic box (10 × 10 × 10 Å^3^) of simple point charge water molecules and followed by energy minimisation with the OPLS3e force field and Broyden-Fletcher-Goldfarb-Shanno algorithms. Later, 100 ns simulations were performed via the isothermal-isobaric (NPT) ensemble at constant pressure (*P* = 1 atm) and temperature (*T* = 300 K) using the Martyna-Tobias-Klein barostat [21] and Nose-Hoover thermostat [22] with 2.0 ps relaxation time. Moreover, the SHAKE scheme was used to constrain all bonds in both the minimisation and MD simulation stages. The long-range electrostatic interactions were described by the Particle Mesh Ewald approach and a cut-off of 9 Å was used to treat the vdWs forces. Finally, 1001 frames from the 100 ns MD trajectory were chosen to evaluate simulation interaction. Trajectory data generated by Desmond was analysed by the simulation interaction diagram. Besides, ligand-protein interaction, root mean square deviation (RMSD) and root mean square fluctuation (RMSF) were analysed to check the stability and residue fluctuation of the interacting complexes. The hydrogen bonds (H-bonds) were identified according to the following conditions: the acceptor-hydrogen-donor angle should be > 135° and the bond length between the acceptor heavy atoms and hydrogen donor should be < 3.5 Å.

### MM-GBSA calculation

Prime MM-GBSA implemented in Prime version 3 module [15] was used to calculate binding free energies (Δ*G*_bind_) for protein-ligand complex by the following Eqs. [7, 23]:

1 $$\Delta {G_{{\rm{bind}}}}\, = \,\Delta {G_{{\rm{complex}}}}\, - \,\left( {\Delta {G_{{\rm{protein}}}}\, + \,\Delta {G_{{\rm{ligand}}}}} \right)$$

2 $$\Delta {G_{{\rm{bind}}}}\,{\rm{ = }}\,\Delta H\, - \,\left( {\Delta {G_{{\rm{solvation}}}}\, + \,T\Delta S} \right)$$

3 $$\Delta {G_{{\rm{bind}}}}\, = \,\Delta {E_{{\rm{MM}}}}\, + \,\Delta {G_{{\rm{GB}}}}\, + \,\Delta {G_{{\rm{SA}}}}\, - \,T\Delta S$$

Here; Δ*G*_complex_, Δ*G*_protein_ and Δ*G*_ligand_ represent the free energies of interacting complexes, protein and ligand in the system, respectively. Δ*G*_bind_ contains VdWs (Δ*E*_vdW_) and electrostatic (Δ*E*_ele_) interactions. Δ*G*_SA_ and Δ*G*_GB_ represent the non-polar and polar contributions of the solvation free energy. Δ*G*_SA_ was estimated by the solvent-accessible surface area (SASA) by the pairwise overlap approach with a probe radius of 1.4 Å, where Δ*G*_SA_ = SASA x 0.0072. Δ*G*_GB_ was evaluated using the generalised Born (GB) model developed by Onufriev and co-workers. *T*Δ*S* is the entropy change based on the ligand binding conformations that were considered in this study due to the low prediction accuracy and high computational cost.

### Expression and purification of *Pf*Hsp70-1

Plasmid expressing *Pf*Hsp70-1 (pQE30/*Pf*Hsp70-1) was used to express recombinant *Pf*Hsp70-1 as previously described [24]. The recombinant *Pf*Hsp70-1 was purified using affinity chromatography as discussed previously but with minor modifications [25, 26]. Triton X (2%) was use to solubilize the protein in lysis buffer (100 mM Tris-HCl, pH 7.5, 300 mM NaCl, 10 mM imidazole, 2% lysis bufer). In this purification method, 1X Sigmafast and urea were not used. Recombinant *Pf*Hsp70-1 protein was further dialysed in PBS buffer (4.3 mM Na_2_HPO_4_, 1.4 mM KH_2_PO_4_, 137 mM NaCl, 3 mM KCl, and 20 mM EDTA; pH 7.4).

### Surface plasmon resonance (SPR) analysis

The steady-state equilibrium binding kinetics of UAA/IMA against PfHsp70-1 were determined using BioBavis Navi 420 A ILVES multi-parametric surface plasmon resonance (MP-SPR) system (Bionavis, Finland) as previously described [18]. Degassed filter sterilised PBS was used as a running buffer. PfHsp70-1 immobilized onto a carboxymethyl dextran (CMD 3-D) gold sensor chip through amine coupling served as ligand. The amount of protein used was just above 200 RU. IMA/UAA were injected at varying concentrations from (0, 62.5, 125, 250, 1000, and 2000 nM) at a 50 µl/min flow rate. Lysozyme was also immobilized onto the same chip as a negative protein control. The interaction was allowed for 8 min at 25 ℃ to determine steady-state equilibrium and then followed by 4 min dissociation. Signals generated were analysed by Data Viewer (BioNavis, Finland). The signal generated by a channel lacking protein-ligand served as a baseline. To determine the equilibrium binding affinities, the resultant sensorgrams were analysed using Trace Drawer software version 1.8 (Ridgeview instrument; Sweden).

## Results and discussion

### Binding pattern and affinity of IMA and UAA inhibitors

**Fig. 1:**
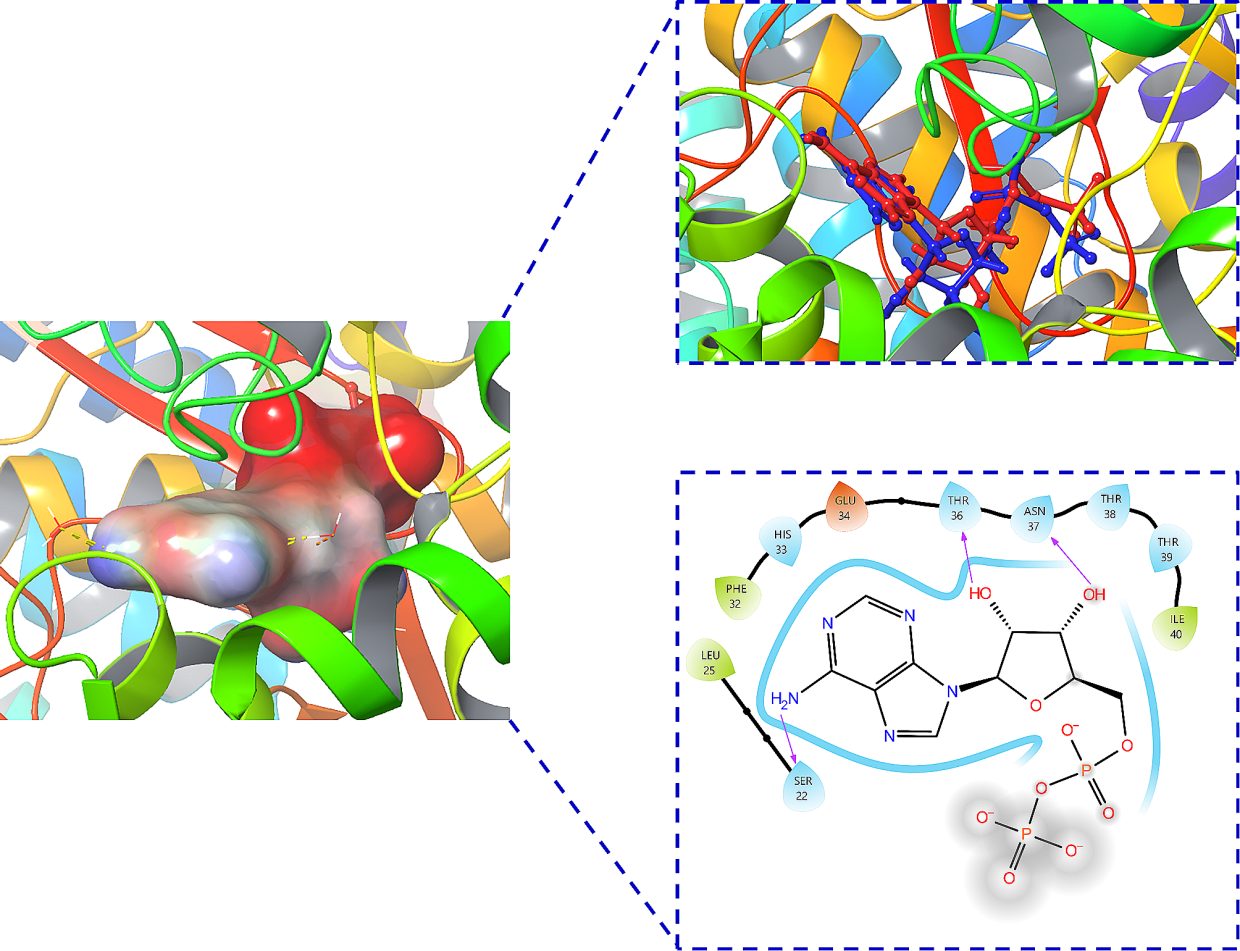
The re-docking result of the re-docked ligand (blue) overlapping with the original ligand (red) in the Hsp70 protein and the 2D interaction diagram

Molecular docking is the most effective program for identifying possible binding modes between proteins [27–30]. For molecular docking to be successful, the Hsp70 protein’s active site must be identified. The accuracy of the docking protocol was examined by re-docking of original ligand in the active site of the Hsp70 enzyme (self-docking) [18, 19, 31]. Before molecular docking, the docking accuracy was verified by re-docking the original ligand (Fig. 1). The redocking approach of the original ligand against the receptor (Hsp70 protein), served as a benchmark for the active site determination. Figure 1 shows the original ligand (red) and re-docked ligand (blue) in almost the same position among the receptor (RMSD = 0.22 Å) that confirmed validation of docking protocol using extra precision glide (XP) scoring function, in the presence of water molecules that are not beyond 5 Å from reference ligand. What’s more, the re-docked ligand is deeply embedded in the pocket of the Hsp70 receptor, and thus produced hydrogen bonds between the amino acids (Ser22, Thr36 and Asn37). The coordinates obtained from the redocking process of the original ligand against the receptor can be employed as a coordinate reference for the docking process of IMA and UAA compounds. The native ligand that has bonded in the spherical cluster is expected to provide precise coordinates based on the ligand reference coordinates of the crystal structure. The interaction between the target Hsp70 protein and the candidate IMA and UAA compounds has been effectively studied using molecular docking. Based on the initial coordinates generated through the redocking procedure utilising flexible conformations, the potential ligand was docked with the Hsp70 protein. Subsequently, IMA and UAA compounds in this study are docked into the Hsp70 receptor to analyse the bonding patterns.

Docking analysis was done to explore the amino acid residues that interact with IMA and UAA compounds, and the active component of Hsp70 protein. Based on the docking results, the docking score is the most accurate approach to define the binding affinity of IMA and UAA complexes.

The docking results suggested that both compounds were held in the binding sites of the *Pf*Hsp70 protein by the combination of several hydrophobic, hydrogen and salt bridge interactions with the *Pf*Hsp70 receptor. The docking results revealed that the highest binding compound to *Pf*Hsp70 protein was IMA with a docking score of -5.388 kcal/mol when compared with UAA (-4.329 kcal/mol), which agreed with the experimentally determined tendency (Table 1). The results of the docking of IMA and UAA ligands with the receptor showed that they had good potential to bind to the 1HSX protein because they had a lower grid score than the candidate protein (Table 1).

**Table 1:** Several scoring functions of IMA and UAA complexes

Inhibitor	Docking score	Glide Gscore	Glide energy	Glide Emodel	Glide Evdw	Glide Ecoul
IMA	-5.388	-5.389	-44.887	-60.839	-33.835	-11.052
UAA	-4.329	-4.335	-46.236	-60.059	-40.897	-5.338
Control						
IMA	-3.557	-3.557	-27.520	-33.969	-21.281	-6.239
UAA	-2.419	-2.428	-23.086	-29.106	-14.358	-8.728

^All the values are in kcal/mol^

**Fig. 2:**
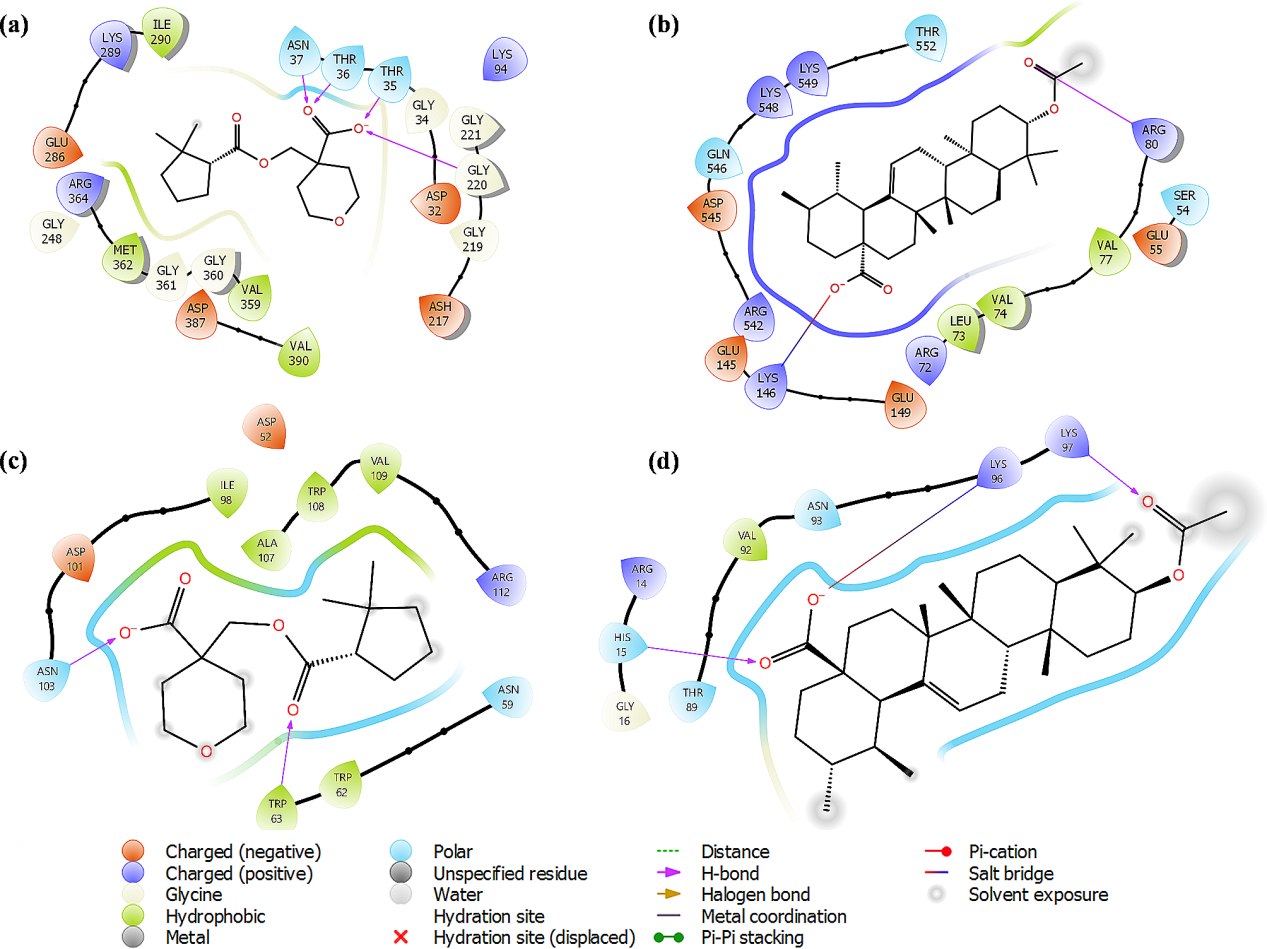
The protein-ligand interactions diagram of (**a** and **b**) Hsp70 and (**c** and **d**) 1HSX receptor with (**a**,**c**) IMA and (**b**,**d**) UAA compounds

However, the glide energy of Hsp70 with IMA compound was slightly smaller than that with UAA. The protein-ligand interaction diagram from docking studies offered in-depth insights into the important amino acid residues, which contributed to the potency of inhibitors (see Fig. 2).

These side chains, particularly those from residues in the exposed cavity, go through conformational change when they engage with ligands at the substrate-binding site. For instance, a significant conformational change occurs in the side chains, which collectively produce a distal subpocket near the exposed cavity. When Lys146 is turned outside the exposed cavity, the distal subpocket enlarges. In the crystal structure of Hsp70, the folded-in conformation of Lys146 decreases the exposed surface of the exposed cavity, allowing a salt bridge interaction with the UAA inhibitor. Although Ser22, Thr 36 and Asn 37 did not directly interact with IMA inhibitors, they serve as the exposed cavity’s tail-cap, creating a more buried protein surface. According to the protein-ligand interactions diagram, the UAA compound formed stable hydrogen bonds with the Arg 80 (1.94 Å) amino acid residue and salt bridge interaction with the Lys 146 (3.10 Å) amino acid residue (Fig. 2b), which could aid in the smooth attachment of the UAA compound into the binding pocket of *Pf*Hsp70 receptor. We observed hydrophobic interactions between Leu 73, Val 74 and Val 77 amino acid residues and UAA compound. Moreover, IMA compound with the highest docking score showed additional hydrogen bonding with the Thr 35 (2.72 Å), Thr 36 (2.03 Å), Asn 37 (1.80 Å) and Gly 220 (1.81 Å) amino acid residues, which could increase the chances of compound possessing good activity (Fig. 2a). IMA compound formed four hydrophobic interactions with Ile 290, Val 359, Met 362 and Val 390 amino acid residues. The protein-ligand interaction results suggest that the UAA-Hsp70 complex had a fewer hydrogen bond formation than the IMA-Hsp70 complex. The 1HSX protein’s molecular docking results showed several types of interactions with the amino acid residues that were on the receptor active site (Fig. 2c **and d**). The IMA ligand shows that two hydrogen bonds linked from residual amino acid residues, including ASN103 and TRP63 to the active site of this pose. Meanwhile, the functional group of the UAA compound shows two hydrogen bond interactions with LYS97 and HIS15 amino acid residues to oxygen atoms of ketone and alcohol carbonyl atoms, respectively. The UAA compound shows that LYS96 amino acid residue is involved in the salt bridge interaction.

### The binding free energy of IMA and UAA inhibitors predicted by MM/GBSA

According to the MD simulations, MM-GBSA binding free energy for the two complexes was evaluated and the result is presented in Table 2.

**Table 2:** Calculated individual energy components and Δ*G*_bind_ predicted by Prime/MM-GBSA method (kcal/mol)

Inhibitor	ΔE_elec_	ΔG_GB_	ΔE_vdW_	ΔG_SA_	ΔG_polar_^a^	ΔG_non−polar_^b^	ΔG_bind_	ΔG_Lipo_
IMA	68.55	-52.38	-35.52	-3.90	16.17	-39.42	-30.26	-13.74
UAA	83.44	-57.94	-45.41	-5.41	25.50	-50.82	-31.19	-11.90
Control								
IMA	60.64	-49.74	-26.78	-1.30	10.9	-28.08	-25.50	-9.17
UAA	66.98	-54.81	-28.65	-1.88	12.17	-30.53	-28.24	-4.93

^a^Δ*G*_polar_ = Δ*E*_elec_ + Δ*G*_GB_; ^b^Δ*G*_non−polar_ = Δ*E*_vdW_ + Δ*G*_SA_

Generally, the binding free energy with more negative values exhibits higher activities of inhibitors [32]. As presented in Table 2, the calculated Δ*G*_bind_ of UAA (-31.19 kcal/mol) was slightly stronger compared with that of IMA (-30.26 kcal/mol). Thus, UAA showed greater inhibitory activity with PfHsp70 than with the IMA compound. This difference was mostly caused by Coulomb interactions, such as the salt bridge between Lys 146 (3.10 Å) and Hsp70 protein. Therefore, UAA is a selective PfHsp70 inhibitor. The binding free energy components suggest that the vdWs forces were the greatest contributors to the Δ*G*_bind_ of both ligands followed by electrostatic energy (Δ*G*_elec_), which suggested that conjugated effects are important for the formation of the protein-ligand complex. Moreover, the influence of conjugated effects on the UAA-Hsp70 complex was greater than the IMA-*Pf*Hsp70 adduct, since the UAA-PfHsp70 complex has a more negative Δ*G*_vdW_ value as compared with the IMA-Hsp70. Moreover, the polar solvation energy is unfavourable for Δ*G*_bind_ since Δ*E*_elec_ values were positive. However, the non-polar solvation energy is favourable because Δ*E*_vdW_ values are negative. However, the IMA ligand could bind more efficiently within the PfHsp70 pocket than the UAA compound, according to the highest lipo energy values. The difference in the non-polar contributions between IMA (-39.42 kcal/mol) and UAA compounds (-50.82 kcal/mol) was 11.40 kcal/mol. Even though the unfavourable polar energy contribution of UAA (25.50 kcal/mol) is higher than that of IMA (15.66 kcal/mol), cannot account for the reduction in the Δ*G*_bind_ induced by the vdWs interactions [33]. The *Pf*Hsp70 complexes show good binding affinity compared to the 1HSX protein. These results indicate that the IMA and UAA compounds have an excellent potential in inhibiting the *Pf*Hsp70 enzyme through a strong binding on the active site of its inhibition. To be more specific, the UAA ligand has a lower Δ*G*_bind_ than the IMA, which allows the UAA ligand as a 1HSX inhibitor to have a better potency than the IMA ligand. Overall, the results show a good correlation with the calculation of the grid score using molecular docking. Ligand that has low binding Δ*G*_bind_ is expected to bind with amino acid residues on the receptor active site, responsible for the activity of the 1HSX protein.

### MD simulation analysis

**Fig. 3:**
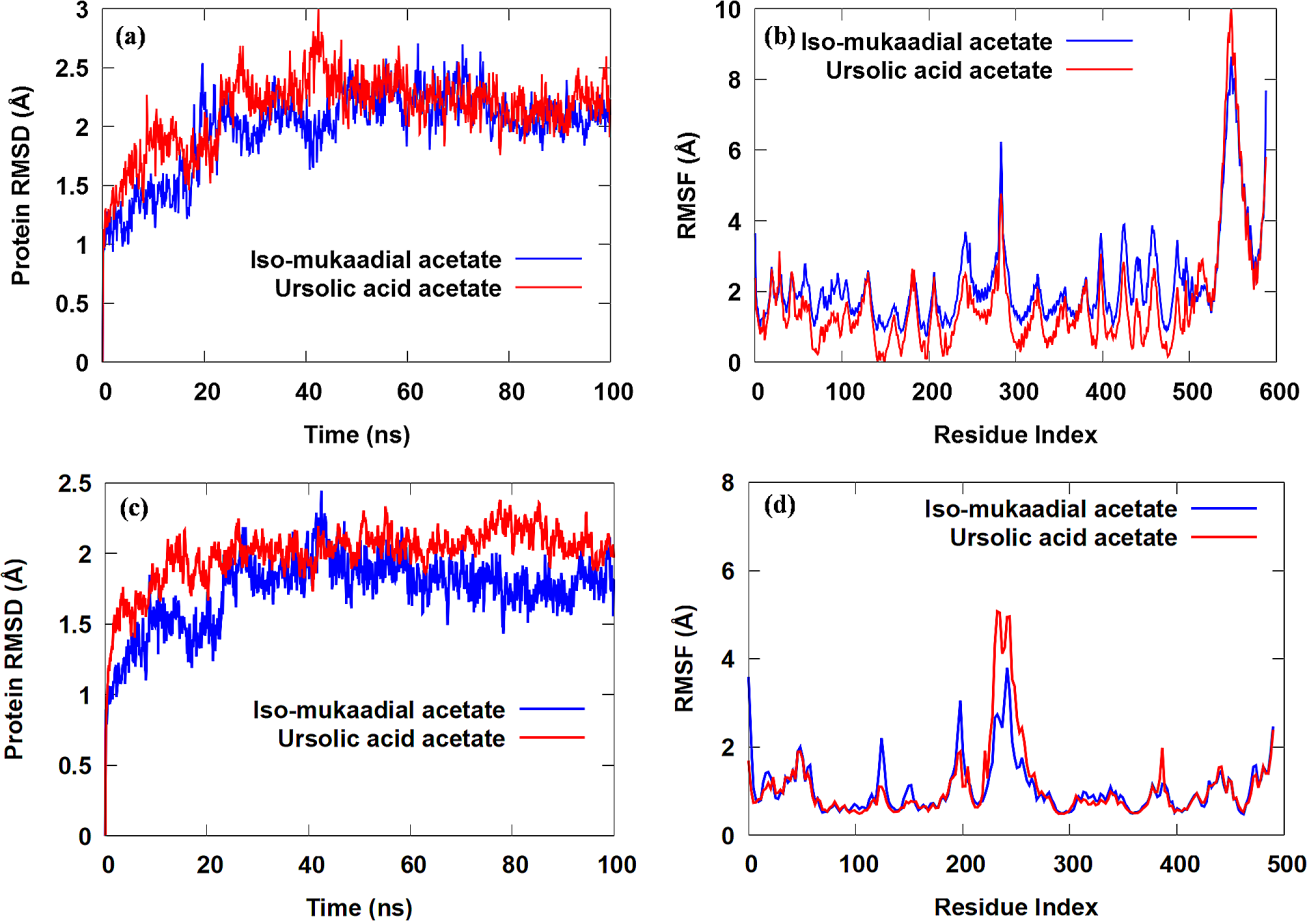
(**a**) RMSD against the dynamics simulation time and (**b**) RMSF against residue number of the Hsp70-ligand complexes. (**c**) RMSD against the dynamics simulation time and (**d**) RMSF against the residue number of the 1HSX complexes

To get deeper structural and energetic of the original ligand and re-docked ligand were considered for MD simulations [18, 31]. The main interactions arising from the docking were not fully maintained after MD runs; we even noted new specific interactions with optimal binding site residue re-organization. MD simulations are regarded as an efficient tool to investigate the dynamics and conformational flexibility changes occurring during protein-ligand binding using the RMSD and RMSF data, see Fig. 3.

Based on the RMSD results in Fig. 3a, both MD simulations attained equilibrium after 45 ns simulations and the system had good convergence. Therefore, the RMSD values of both complexes showed consistent and stable behaviour after 45 ns at RMSD values (1.79 to 2.71 Å and 1.43 to 2.23 Å for IMA-Hsp70 and UAA-*Pf*Hsp70 complexes, respectively). Based on the results shown in Fig. 3a, both RMSD fluctuations have similar changing trends, suggesting that the MD simulations studies on IMA-PfHsp70 and UAA-Hsp70 complexes are reasonable.

The fluctuations of the protein amino acid residues, stability changes of the identical residue, and the influence of inherent local conformational flexibility on the receptors were explored by measuring the RMSF (Fig. 3b). The RMSF trends of fluctuations and distributions of IMA-*Pf*Hsp70 and UAA-PfHsp70 complexes suggest that the binding patterns of both inhibitors are almost identical. According to the RMSF plot, amino acid residues of IMA-*Pf*Hsp70 complex fluctuated more significantly than amino acid residues in UAA-Hsp70 complex, signifying that the IMA inhibitor could form strong interactions with *Pf*Hsp70. Moreover, key amino acid residues, including Asp 32, Thr 35, Thr 36, Asn 37, Lys 94, Leu 218, Gly 220, Gly 221 and Gly 360 formed strong H-bonds interactions with IMA inhibitor. Moreover, analysis of the RMSF of the key amino acid residues (i.e., Asn 53, Leu 73, Lys 146 and Asn 556) involved in the formation of H-bonds in UAA-Hsp70 complex.

Clearly from Fig. 3c, the RMSD values of IMA and UAA compounds fitting on the 1HSX protein Cα fluctuated within the ranges of 1.5–2.4 Å and 1.7–2.3 Å after 70 and 80 ns stabilized, respectively, and finally converged at approximately 2 Å. Compared to the UAA, the RMSD of the IMA tended to equilibrate after 22 ns, which indicated that UAA is more active than IMA in the 1HSX receptor. Analysis showed that the fluctuation of RMSD value in the amino acid residues in both systems is less than 3 Å, thus indicating that the binding of IMA and UAA compounds to 1HSX protein was relatively stable during the MD simulations. Furthermore, the range of fluctuation in the RMSD value of UAA was relatively lower than that of the IMA, thus, demonstrating that UAA formed a relatively firm interaction in the active sites of 1HSX. Figure 3d shows the relationship between the 1HSX protein complexes’ RMSF value and a number of residues. The RMSF distributions and dynamic fluctuation patterns of the IMA and UAA complexes were comparable, indicating that the binding of these inhibitors to 1HSX was similar. Additionally, it was demonstrated that the majority of protein residues in each complex had RMSF values that were lower than 4 Å. Moreover, it was found that the RMSF fluctuations in the IMA complex were lower than those in the UAA complex, suggesting that it had less structural mobility than the UAA. These findings revealed that the binding affinities of IMA and UAA to 1HSX protein were generally favourable.

**Fig. 4:**
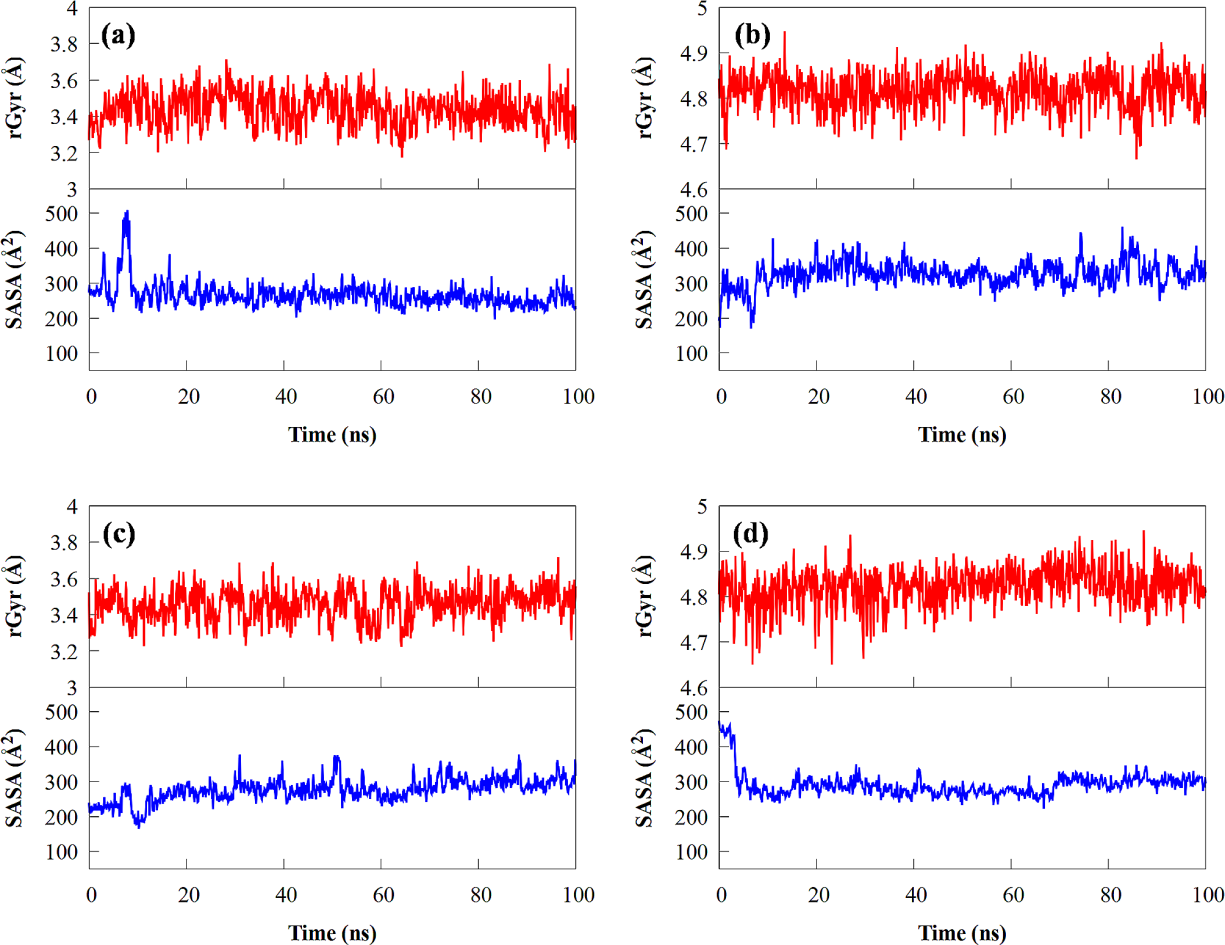
The radius of gyration (above), and the solvent-accessible surface area (below) of (**a** and **b**) Hsp70 and (**c** and **d**) 1HSX receptor with (**a**, **c**) IMA and (**b**, **d**) UAA compounds

The density of a protein structure may be determined using the radius of rotation (*R*_g_), which is dependent on how close an atomic mass is to the centre of gravity of a particular molecule. Additionally, one of the factors for analysing the compactness of complex structures is the *R*_g_ analysis. The *R*_g_ value can give a clear picture of an unfolded or steadily folded structure. A higher *R*_g_ number denotes a dynamic simulation in which the system expands. Figure 4 clearly shows changes in the *R*_g_ within the four systems in the 100 ns MD simulations. Additionally, 100 ns was used to determine the *R*_g_ of a protein with a ligand in the active sites. The best-docked model’s *R*_g_ values began at 3.2 Å in the MD simulations, and the structure steadily expanded and shrunk within the limit. *R*_g_ remained almost the same throughout the process. Even though during the 26–59 ns, the *R*_g_ value increased from 3.2 to 3.7 Å and subsequently reduced to 3.65 Å, returning the system to a position close to its initial position, it was clear that the receptor-ligand remained stable and firmly packed. Moreover, the *R*_g_ of UAA steadily decreased to stability before converging around 4.65 to 4.95 Å. This demonstrated that UAA significantly affects the density of protein structures. The results show that the mean *R*_g_ values of the PfHsp70 complexes are relatively identical and consistent with the mean *R*_g_ values of 1HSX. The optimum docking position closely matched the reference pattern based on the *R*_g_ values obtained from MD simulations for the protein’s compactness. Thus, these results identify the folded PfHsp70 structure as stable.

The solvent-accessible surface area (SASA) parameter measures the number of water molecules that can interact with the complex’s active site surface during the simulation. The results showed no significant difference in the mean value of SASA (Å^−2^) between the 1HSX protein and PfHsp70 complex (Fig. 4). For each complex, the SASA values’ overall analysis revealed good stability. Low SASA value fluctuation is evidence of it.

**Fig. 5:**
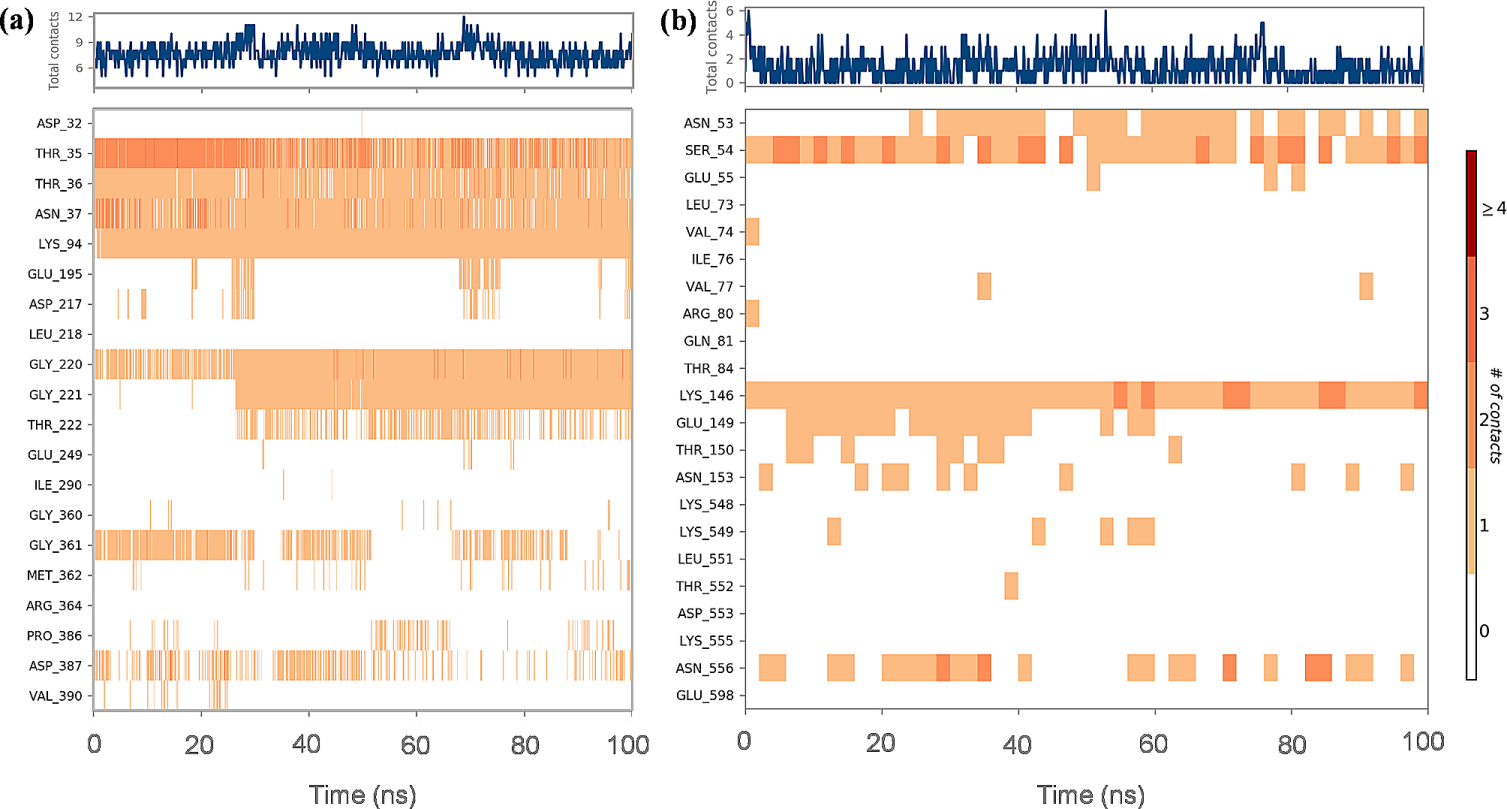
Protein–ligand contact timeline for PfHsp70 protein complexed with (**a**) IMA and (**b**) UAA compound

Hydrogen bonds (H-bonds) are essential for ligand binding. Because hydrogen-bonding characteristics have such a significant impact on drug selectivity, metabolization, and adsorption, they must be considered while developing new drugs. H-bond distance analysis was carried out on each complex to estimate the H-bond interactions. A timeline representation of interactions and contacts (H-bonds, hydrophobic, ionic, and water bridges) is summarized in Fig. 5. The top panel shows the total number of specific interactions the PfHsp70 protein makes with the ligand over the 100 ns MD simulation trajectory. The bottom panel shows which residues interact with the ligand in each trajectory frame. According to the scale to the right of the figure, certain residues have many specific contacts with the ligand, which is depicted by a deeper orange colour. In the MD simulation process, 1 > 2 > 3 hydrogen bonds make up the bulk of protein-ligand complexes, with 4 hydrogen bonds being the least common. The PfHsp70 protein complexed with IMA and UAA compounds could form a maximum 3–4 hydrogen bonds with active site residues. Fluctuations in the number of hydrogen bonds are relatively stable, indicating that the system is in a stable state. The equal number of hydrogen bonds, most of which are hydrogen bonds 1 to 3, helps the ligand remain stable by preventing rapid changes in the hydrogen bonds. This further proved stable interactions formed between compounds and proteins in the equilibrium state. According to these findings, hydrogen bonds are essential to the conformational stability of all four complexes.

**Fig. 6:**
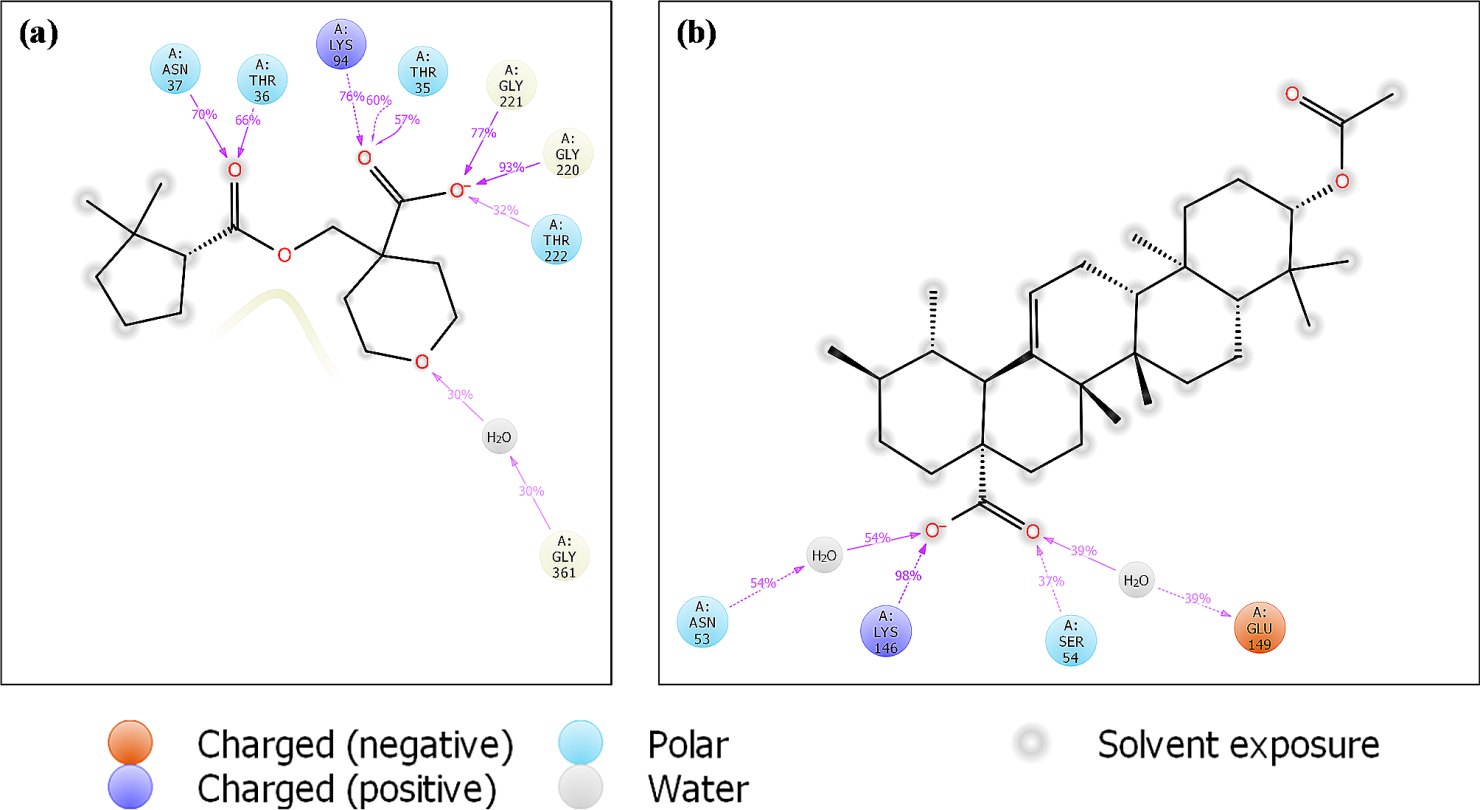
Two-dimensional protein-ligand interactions of (**a**) IMA-Hsp70 and (**b**) UAA-Hsp70 complexes after MD simulations

We observed that PfHsp70 Thr 35, Thr 36, Asn 37, Lys 94, Gly 220, Gly 221 and Thr 222 amino acid residues formed stable H-bonds with IMA (Fig. 6a), while Gly 361 residue could interact with IMA residues through a water molecule by forming strong H-bonds.

Residues Lys 146 contributed greatly to UAA inhibitor with the interaction fractions up to 0.98 (Fig. 6b). H-bonds interaction was established between UAA inhibitor and residues Ser 54, Asn 53 and Glu 149 with the last two doing so with the help of water molecules.

### Prediction of absorption, distribution, metabolism, and excretion (ADME) properties

Before conducting in vivo testing, it is crucial to examine the pharmacokinetic properties of any molecule being considered as a potential drug candidate, including factors such as absorption, distribution, metabolism, excretion, and toxicity (ADME). The drug-likeness of synthesized compounds is predicted by analysing their ADME properties based on Lipinski’s rule of five. These essential parameters not only determine the similarity of the substance to a drug, but also its effectiveness within the body. ADME analysis provides an important image in predicting the activity of the candidate as a drug before using molecular docking against the PfHsp70 enzyme. ADME descriptors of IMA and UAA ligands were calculated using the QikProp v6.0 module in Maestro 12, Schrödinger 2019-2 [34]. Moreover, the studied compounds were investigated based on thirty-four (34) pharmaceutically relevant properties and their capabilities to bind *Pf*Hsp70. According to these ADME properties, such as % human oral absorption, log P MDCK, log *P* (octanol/water), H-bond donor, H-bond acceptor, molecular volume, molecular weight, and their position according to Lipinski’s rule of 5, which describes molecular properties vital for the pharmacokinetic profile of drugs in living systems [34] are summarised in Table 3.

**Table 3:** The predicted principal descriptors and physiochemical descriptors for IMA and UAA compounds towards the PfHsp70 receptor

Principal descriptors	IMA	UAA	Range 95% of Drugs
Carbon Pi SASA	0	7.926	0.0–450.0
Dipole moment (D)	4.321	4.446	1.0–12.5
Electron affinity (eV)	-0.904*	-0.967*	-0.9–1.7
Globularity (Sphere = 1)	0.882	0.852	0.75–0.95
Hydrogen bond acceptor	5.7	4	2.0–20.0
Hydrogen bond donor	1	1	0.0–6.0
Hydrophilic SASA	95.513	103.854	7.0–330.0
Hydrophobic SASA	432.805	639.688	0.0–750.0
Ionization potential (eV)	10.905*	9.426	7.9–10.5
Molecular volume (Å^3^)	945.841	1523.596	500.0–2000.0
Molecular weight	284.352	498.745	130.0–725.0
Number of rotatable bonds	4	2	0.0–15.0
Total SASA	528.317	751.468	300.0 -1000.0
vdW polar SA (PSA)	82.642	75.052	7.0–200.0
Weakly polar SASA	0	0	0.0–175.0
Predictions for properties			
% Human oral absorption	88	100	< 25% is poor
Apparent Caco-2 permeability (nm/sec)	311	259	< 25 poor, > 500 great
Apparent MDCK permeability (nm/sec)	178	146	< 25 poor, > 500 great
HERG K + channel blockage: log IC50	-1.633	-2.155	concern below − 5
Jm, max transdermal transport rate	0.194	0	micrograms-cm^2^-hr
Jorgensen Rule of 3 violations	0	1	maximum is 3
Lipinski Rule of 5 violations	0	1	maximum is 4
Number of primary metabolites	2	2	1.0–8.0
QP log BB for brain-blood	-0.496	-0.511	-3.0–1.2
QP log K hsa Serum Protein Binding	-0.209	1.771*	-1.5–1.5
QP log Kp for skin permeability	-2.896	-3.214	Kp in cm-hr
QP log P for hexadecane-gas	8.015	13.199	4.0–18.0
QP log P for octanol-gas	13.747	21.568	8.0–35.0
QP log P for octanol-water	2.723	7.029*	-2.0–6.5
QP log P for water-gas	7.837	7.252	4.0–45.0
QP log S - conformation independent	-2.765	-7.823	-6.5–0.5
QP log S for aqueous solubility	-3.27	-8.168*	-6.5–0.5
QP polarizability (Å^3^)	29.130	53.612	13.0–70.0
Qualitative model for human oral absorption	High	low	> 80% is high

^*indicates a violation of the 95% range^

The Lipinski rule, also known as the rule of five, utilizes basic molecular descriptors formulated to determine drug likeness. According to this rule, most drug-like molecules possess a Log P value less than or equal to 5, a molecular weight less than or equal to 500 Da, and no more than 10 hydrogen bond acceptors and 5 hydrogen bond donors. Molecules that violate more than one of these criteria may face challenges with bioavailability. Studied IMA and UAA compounds have molecular weight < 500. Low molecular weight drug molecules (< 500) are easily transported, diffused, and absorbed as compared to heavy molecules. Molecular weight is an important aspect of therapeutic drug action, if it increases correspondingly, it affects the drug action. A number of hydrogen bond acceptors and number of hydrogen bond donors in the tested compounds were found to be within the Lipinski limit. The log Kp values ranged from − 3.093 to -2.896, suggesting that the skin permeability of the designed compounds is better. Cell permeability (QPPCaco), which is used to access cell permeability in biological membranes and is a key factor governing drug metabolism ranged from 259 to 311, whereas pMDCK (cell-permeable parameter) values ranged between 146 and 178. Moreover, both compounds showed a good partition coefficient (QPlogPo/w) (2.723 to 7.029) that is critical to the distribution and absorption of drugs within the human body. The drugs are usually mostly taken in oral formulations, which must be absorbed by the intestine to exert their effects. The % human oral absorption for both inhibitors ranged between 88 and 100%, while their *p* log HERG (K + channel blockage) data in the range of -1.633 to -2.155 were less than − 5, water solubility (QP log *S*) ranged from − 3.270 to -8.244 and skin permeability (log *K*_p_) data from − 2.896 to -3.183 were all within the acceptable range. The QP log BB parameter, which indicates the capability of a drug to pass via the blood-brain barrier was within the acceptable range. More importantly, the results obtained by QP log *P*_o/w_, QP log HERG, QPPCaco, and human oral absorption showed that the compound has the advantages of high solubility, low cardiotoxicity, good membrane permeability, and oral absorption. For toxicity assessment, all compounds were negative for AMES values and skin sensitization indicators, and tests showed that the compounds were not mutagenic and did not cause skin sensitization. The carcinogenicity was also shown to be negative, which did not cause mutations in the organism to the compounds that have some safety for the organism. These results indicated that the compounds exhibited good characteristics in intestinal absorption, distribution volume, and toxicity, presented high biological activities, and therefore potentially interesting candidates for further studies. Overall, the results indicated that the IMA inhibitor met the criteria for a good ADME as a drug. Especially the toxicity parameter, each candidate shows good suitability as a drug because it is non-toxic, and therefore, is more likely to be developed as a therapeutic molecules. To further investigate the pharmacokinetic properties of the compounds, the therapeutic effects of the compounds on model mice need to be studied by constructing a mouse model of hyperuricemia.

### SPR analysis SPR kinetics data for the interaction of *Pf*Hsp70 with either IMA or UAA

**Fig. 7:**
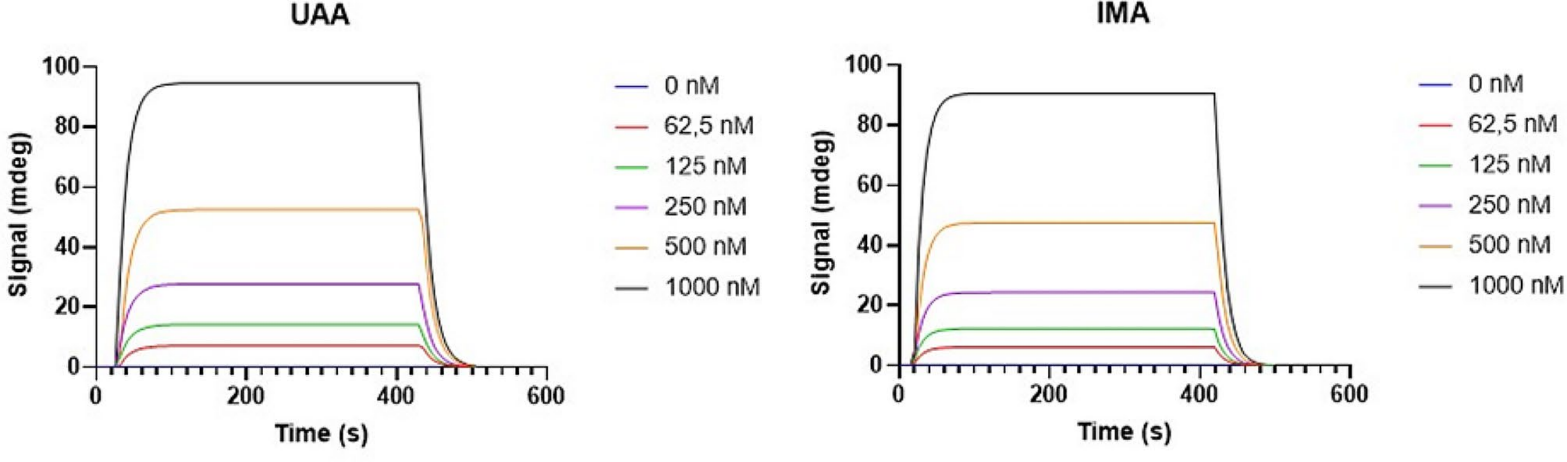
SPR sensorgrams for *Pf*Hsp70 binding to UAA/IMA

We further conducted SPR analysis to explore the direct binding of UAA and IMA (Fig. 7). The findings demonstrate that PfHsp70 binds to either of the two compounds within the micromolar range of affinity (Table 4). The SPR data suggest that PfHsp70 exhibits modest affinity for the two compounds. We generated a flat graph for SPR with both compounds on assaying with lysozyme (Supplementary Figure S1). This validates that none of the two compounds interacts with lysozyme as a non-Hsp70 control. The data validates that the interaction of PfHsp70-1 with the two compounds is specific and this is in agreement with the data obtained from the *in-silico* findings. Furthermore, the findings suggest that PfHsp70 is capable of directly binding each of the two compounds. However, our current findings do not rule out the prospect of the compounds binding to other targets apart from PfHsp70 (Fig. 7).

**Table 4:** SPR kinetics data for the interaction of *Pf*Hsp70 with either IMA or UAA

Compound	Ka	Kd	KD	Chi^2^	U-Value
IMA	1.72E + 04 ± 7,52e2	6.98E-02 ± 9,01e-6	9.89E-06 ± 1,39e-6	7.56	7.7
UAA	1.59E + 04 ± 1,93e3	6.41E-02 ± 1,57e-5	4.03E-06 ± 4,97e-7	8.02	7.1

## Conclusion

In this study, MM/GBSA, MD and molecular docking simulations were used to investigate the binding and selectivity mechanisms of IMA and UAA compounds towards the *Pf*Hsp70 receptor. The MM/GBSA analysis suggests that vdWs interactions play a key influence in the binding free energies of IMA and UAA compounds in the *Pf*Hsp70 binding pocket. Moreover, the protein-ligand interactions diagram reveals hydrogen bonding and hydrophobic interactions in enhancing the binding affinity and stability of the inhibitor at the pocket site. In the current study, we established that both IMA and UAA inhibit *Pf*Hsp70 chaperone function. Furthermore, the predicted ADME properties in the acceptable range for both inhibitors suggested that they are a drug-like candidate. The *in-silico* studies offer insights into the structural features of IMA and UAA inhibitors and their interaction with *Pf*Hsp70. Altogether, our findings add UAA and IMA to the growing list of known *Pf*Hsp70 inhibitors.

### Electronic supplementary material

Below is the link to the electronic supplementary material.

Supplementary Material 1

## Data Availability

Data is provided within the manuscript or supplementary information files.
