# Supplementary material for: Iso-mukaadial acetate and ursolic acid acetate bind to Plasmodium Falciparum heat shock protein 70: towards targeting parasite protein folding pathway
Source: BMC Chem. 2024 Mar 18;18(1):55. doi: 10.1186/s13065-024-01159-6 (PMC10949600; doi:10.1186/s13065-024-01159-6)
Supplement: Supplementary file 1 — Supplementary Material 1 [file 13065_2024_1159_MOESM1_ESM.docx]

**Supplementary figure**

S1: SPR sensorgrams for IMA (analyte) injected into immobilized lysozyme (ligand).

**
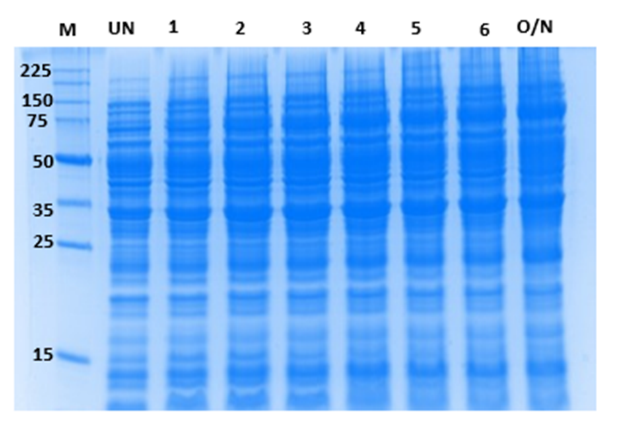

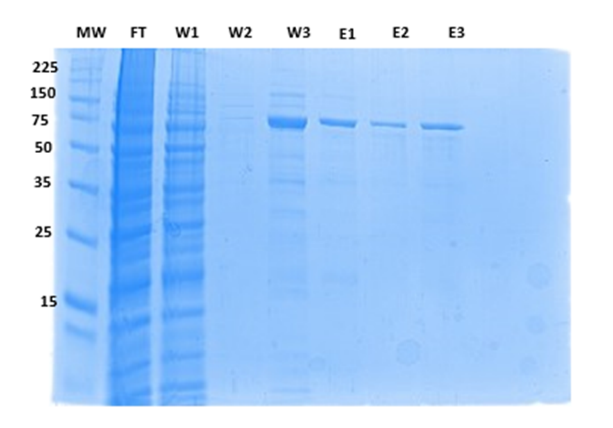
**

S2**:** Recombinant expression and purification analysis of PfHsp70-1 expressed in *E. coli* BL21 (DE3) cells using 0.5 mM IPTG and purified using nickel affinity chromatography. Lane M: Protein marker, UN: Uninduced cells (control), lane 1-6: hourly samples taken post expression induction, lane O/N: Overnight expression, lane: FT: Flow through, lane W1-W3: Wash 1-3, and lane E1-E3: Elution 1-3.
